# Exploratory genetic analysis in children with autism spectrum disorder and other developmental disorders using whole exome sequencing

**DOI:** 10.17305/bb.2024.10221

**Published:** 2024-08-01

**Authors:** Edin Hamzic, Lemana Spahic, Nirvana Pistoljevic, Eldin Dzanko, Sanela Pasic, Lejla Kadric, Fadila Serdarevic, Aida Hajdarpasic

**Affiliations:** 1 Biocomputix, Sarajevo, Bosnia and Herzegovina; 2 BioCertica, Paarl, South Africa; 3 International Burch University, Sarajevo, Bosnia and Herzegovina; 4 Education for All (EDUS), Sarajevo, Bosnia and Herzegovina; 5 Department of Economics and Business, Sarajevo School of Science and Technology, Sarajevo, Bosnia and Herzegovina; 6 Department of Medical Biology and Genetics, Sarajevo Medical School, Sarajevo School of Science and Technology, Sarajevo, Bosnia and Herzegovina; 7 Department of Epidemiology, Sarajevo Medical School, Sarajevo School of Science and Technology, Sarajevo, Bosnia and Herzegovina; 8 Department of Child and Adolescent Psychiatry, Erasmus MC, Rotterdam, The Netherlands

**Keywords:** Autism spectrum disorder (ASD), developmental disorders (DDs), whole exome sequencing (WES), genes

## Abstract

Developmental disorders (DDs), such as autism spectrum disorder (ASD), incorporate various conditions; once identified, further diagnostics are necessary to specify their type and severity. The aim of this exploratory study was to identify genetic variants that can help differentiate ASD early from other DDs. We selected 36 children (mean age 60.1 months) with DDs using Developmental Behavioral Scales (DBS) through “EDUS-Education for All”, an organization providing services for children with DDs in Bosnia and Herzegovina. We further rated children’s autistic traits with the preschool version of the Childhood Autism Rating Scale, second edition (CARS-II). We defined ASD if scores were >25.5 and other DDs if scores were <25.5. Diagnosis of ASD and DD were independently confirmed by child psychiatrists. Whole exome sequencing (WES) was performed by Veritas Genetics, USA, using Illumina NovaSeq 6000 (Illumina Inc., San Diego, CA, USA) NGS sequencing apparatus. We tested genetic association by applying SKAT-O, which optimally combines the standard Sequence Kernel Association Test (SKAT) and burden tests to identify rare variants associated with complex traits in samples of limited power. The analysis yielded seven genes (*DSE*, *COL10A1*, *DLK2*, *CSMD1*, *FAM47E*, *PPIA*, and *PYDC2*) to potentially differentiate observed phenotypic characteristics between our cohort participants with ASD and other DDs. Our exploratory study in a small sample of participants with ASD and other DDs contributed to gene discovery in differentiating ASD from DDs. A replication study is needed in a larger sample to confirm our results.

## Introduction

Developmental disorder (DD) incorporates various conditions, such as language disorder, learning disorder, or autism spectrum disorder (ASD). It represents the joint initial diagnosis preschoolers are given when a norm-based developmental delay in at least two main developmental areas is reliably observed and measured [1]. After initially identifying a DD, further diagnostic efforts are needed to specify its type and severity. It is especially effortful to diagnose ASD, a DD characterized by communication difficulties, social communication and interaction impairments, and repetitive and restricted behavioral patterns ranging from mild to severe forms [2]. Most recent studies have shown that prevalence rates of ASD have increased significantly during the last few decades and are currently as high as 2% [3, 4]. A typical clinical approach for diagnosing ASD is applying standardized clinical observational rating scales and interviews, usually before the age of three [5–7]. Despite a broad scientific and practical justification of the use of observational schedules and scales, various limitations result in the underidentification of children with ASD worldwide. In low- to mid-income countries, for example, where it has been estimated that around half of children with disabilities in the world live, the lack of reliable screening tools and trained professionals leaves most children with ASD undiagnosed [8, 9]. Therefore, the differentiation between general DD and ASD represents a great challenge for most low- to mid-income countries’ health, educational, and social systems, mainly because ASD trajectories differ significantly from other DD [10, 11]. Parents of children with moderate and severe ASD in Bosnia and Herzegovina reported a delay in their child’s development, on average at 18 months, yet not receiving a precise ASD diagnosis [12]. Even after an average of five medical professional visits and, on average, 18 months later, they still receive DD diagnosis only [12]. In addition, a gender bias in diagnosing ASD provides recent insights that the traditional approaches in diagnosing ASD are not sex/gender sensitive enough and leave preschool-aged girls often undiagnosed because of possible cultural confounds [13]. The differentiation between ASD and other DDs still presents a great challenge and often depends on other factors, such as income or sex/gender, leaving the scientific community with the task of finding the additional missing puzzle pieces for “early, valid, and reliable diagnosis of ASD for all” [14].

The evolution of genetics and its diagnostics, based on genetic variants detection and gene expression profiling, broadened the diagnostic horizons and enabled medical experts to confirm diagnoses for which biomarkers are already established [15]. While the line of differentiation is so fine that finding one between ASD and another DD is both extremely difficult and essential, genetics may contribute significantly [16]. Most recent ASD studies show that it is often a comorbid disorder with intellectual disability, epilepsy, motor dysfunction, and anxiety [17]. Fragile X syndrome, tuberous sclerosis, Rett syndrome, and neurofibromatosis can occur in the syndromic form of ASD; however, ASD can also occur as a non-syndromic disease [17]. Rare microdeletions or single gene defects occurring together with ASD are Williams-Beuren, Cowden, Sotos, Moebius Smith–Lemli–Opitz, Timothy syndromes, and untreated phenylketonuria [17]. Multifactorial genetics behind DDs is pronounced in ASD, with hundreds of genes implicated in its onset either directly or indirectly. Computational psychiatry in the Precision Medicine Initiative emphasizes the importance of an objective and personalized approach to healthcare using combinations of knowledge layers leading to a tailored examination of disorder progression, treatment options, and prognosis [14].

This increasing complexity when diagnosing ASD emphasizes the need to develop functional diagnostic panels. One approach would be identifying genetic factors differentiating ASD from other DDs. For this purpose, through this exploratory study, we performed differentiation analysis in these two populations using the whole exome sequencing data (WES). Regarding DDs and their complexity, whole genome sequencing (WGS) or WES strategies yield the most comprehensive results. While WES is focused on detecting genes implied in some function or disorder, WGS aims to detect genetic changes that affect the activity of the genes and protein production, leading to various genetic disorders [18]. Determining all genetic factors involved in developmental disorders is indeed a path to elucidate underlying mechanisms and potentially discover preventive and treatment options. Elucidation of the genetically based differences between ASD and other DDs would serve as a basis for further diagnostic development in the field of ASD. Efficient genetic tools would aid in the early diagnosis of ASD and, therefore, timely intervention.

In light of these gaps, we performed an exploratory pilot study examining genetic-based variability between ASD and other DDs in a small Bosnian and Herzegovinian cohort of children with DDs.

## Materials and methods

### Participants and assessment

We selected children with DDs using Developmental Behavioral Scales (DBS) through “EDUS-Education for All”, a non-governmental organization providing services for children with various DDs and their families in collaboration with the public educational system in Bosnia and Herzegovina [19, 20]. Participants of this study, as children with identified atypical development, were regularly enrolled in the EDUS preschool program and received intensive early intervention based on the Comprehensive Application of Behavior Analysis (CABAS^®^) system. CABAS^®^ is a research program and evidence-based behavioral approach to assessing and educating children with and without DDs, focusing on children diagnosed with ASD. Thousands of children across the country are enrolled in the EDUS preschool programs, receiving developmental screening, detailed assessment, behavioral and educational treatments, and parental education services [21–24]. We selected children for our study using the DBS [23]. The DBS consists of ten age-appropriate developmental screening scales for children from one month to six years of age covering all five developmental areas: speech and communication, motor development (gross and fine), cognitive development, social–emotional development, and self-help/adaptive skills. The primary function of the DBS is to detect atypically developing children, which is achieved when comparing developmental area results to the typical developmental norms for the particular chronological age in Bosnia and Herzegovina. Children whose DBS results indicated a developmental delay (atypical development) in at least one developmental area were included in the study and constituted the group of children with a DD.

All participants were divided into two groups: 15 participants with other DDs and 25 participants with ASD (Table 1).

**Table 1:** Cohort characteristics

**Characteristics**	**Developmental disorder**	**Autism spectrum disorder**
*Gender*		
Male	11 (85.0%)	17 (74.0%)
Female	2 (15.0 %)	6 (26.0%)
Total	13 (100.0%)	23 (100.0%)
*Mean age of participants (months)*		
Male	62.2 (SD ═ 11.3, range 46–79)	59.7 (SD ═ 16.7, range 24–85)
Female	60.50 (SD ═ 6.4, range 56–65)	57.33 (SD ═ 9.6, range 47–65)
Total	61.9 (SD ═ 10.5, range 46–79)	59.1 (SD ═ 15.0, range 24–85)
*Mean gestational age at delivery (weeks)*		
Male	38.2 (SD ═ 3.1, range 30–41)	38.6 (SD ═ 1.8, range 34–41)
Female	39 (SD ═ 1.4, range 38–40)	38.3 (SD ═ 1.4, range 36–40)
Total	38.3 (SD ═ 2.9, range 30–41)	38.5 (SD ═ 1.6, range 34–41)
*Mean child birth weight (grams)*		
Male	3325.4 (SD ═ 810.0, range 1580–4500)	3336.2 (SD ═ 565.4, range 1850–4100)
Female	2950.0 (SD ═ 565.7, range 2550–3350)	3128.3 (SD ═ 414.5, range 2650–3750)
Total	3267.7 (SD ═ 770.3, range 1580–4500)	3282.0 (SD ═ 529.4, range 1850–4100)
*Mother’s age at delivery (years)*		
**Male**		
18–30	7	11
31–40	4	6
**Female**		
18–30	0	4
31–40	2	2
*Father’s age at delivery (years)*		
**Male**		
18–30	3	4
31–40	7	12
40+	1	1
**Female**		
18–30	0	3
31–40	2	3
*Detected developmental delay*		
1 developmental area	0 (0.0%)	0 (0.00%)
2 developmental areas	0 (0.0%)	0 (0.00%)
3 developmental areas	2 (13.3%)	1 (4.0%)
4 developmental areas	3 (20.0%)	2 (8.0%)
5 developmental areas	10 (66.7%)	22 (88.0%)
*Clinical opinion from child psychiatrist*		
Male observed	5	11
Female observed	1	4
Mean age	66.0 (SD ═ 3.5, range 60–70)	64.3 (SD ═ 13.9, range 48–90)
Concluded ASD	0	15
Concluded other DDs	6	0
Sample covered	6 (40.0%)	15 (60.0%)

ASD: Autism spectrum disorder, DDs: Developmental disorders.

We further rated children’s autistic symptoms using Bosnian–Croatian–Serbian translated and publisher-approved version of the Childhood Autism Rating Scale, second edition (CARS-II), a clinical rating scale for identifying ASD in children aged two years and above through direct behavioral observation [12, 25, 26]. We defined ASD if scores were >25.5 and other DDs if scores were <25.5 [12]. To get more clarity and ensure additional validity of group selection, we conducted the third step consisting of clinical observations by an experienced child psychiatrist in the field of ASD. The child psychiatrist observed children in their natural environment, collected information on the developmental histories of children from the files, and, based on that information, categorized them as diagnosed with ASD or other DDs. The child psychiatrist was blinded to the CARS-II scores of participants during the diagnostic process. Clinical observations showed 100% concordance between child psychiatrists’ observations and CARS-II classification and results (cut-off score 25.5) [12].

### DNA isolation, library preparation, and WES

DNA was isolated from 40 participants’ saliva samples using the Samplify DNA collection kit and following the manufacturer’s protocol (OraSure Technologies Inc., Ottawa, Ontario, Canada). For each sample, 20-µL nuclease-free water is added to one well of a 96-well PCR plate. Heat-treated Samplify DNA collection tube is vortexed, and 30 µL of saliva is transferred from the tube to the well with water and slowly pipetted to mix. Sample purification beads (SPBs) were vortexed and inverted a few times to resuspend. At the end of this process, genomic DNA is extracted.

Library preparation for WES was performed using Nextera DNA Exome kit (Illumina Inc., San Diego, CA, USA), previously known as TruSeq Rapid Exome Kit. This kit is optimized to provide 45 Mb coverage of exomes in a uniform and specific manner, enabling comprehensive exome sequencing. Detailed library preparation protocol is provided in Supplementary methods 1.

**Table 2:** Genetic association test results (significance level *P* ═ 1×10^-4^)

**Gene**	**Ensembl ID**	**Description**	***P*-values**
*DSE*	ENSG00000111817	dermatan sulfate epimerase [Source: HGNC Symbol; Acc: HGNC:21144]	5.63×10^-05^
*COL10A1*	ENSG00000123500	collagen, type X, alpha 1 [Source: HGNC Symbol; Acc: HGNC:2185]	5.66 × 10^-04^
*DLK2*	ENSG00000171462	delta-like 2 homolog (Drosophila) [Source: HGNC Symbol; Acc: HGNC:21113]	4.90×10^-04^
*CSMD1*	ENSG00000183117	CUB and Sushi multiple domains 1 [Source: HGNC Symbol; Acc: HGNC:14026]	9.76×10^-04^
*FAM47E*	ENSG00000189157	family with sequence similarity 47, member E [Source: HGNC Symbol; Acc: HGNC: 34343]	4.92×10^-04^
*PPIA*	ENSG00000196262	peptidylprolyl isomerase A (cyclophilin A) [Source: HGNC Symbol; Acc: HGNC:9253]	2.29×10^-04^
*PYDC2*	ENSG00000253548	pyrin domain containing 2 [Source: HGNC Symbol; Acc: HGNC:33512]	1.16×10^-04^

WES was performed by Veritas Genetics, USA, using Illumina NovaSeq 6000 (Illumina Inc., San Diego, CA, USA) NGS sequencing apparatus. The whole exome sequencing protocol is detailed in Supplementary methods 2.

### Sequence alignment and variant calling

Variant calling was performed using FreeBayes (version v0.9.21-24-g840b412-dirty), including duplicate marked alignments in the analysis and with all other parameters set on default values. FreeBayes is a haplotype-based variant detector for small variations, such as single-nucleotide polymorphisms and insertions or deletions, multi-nucleotide polymorphisms, and complex events (composite insertion and substitution events) smaller than the length of a short-read sequencing alignment [27]. FreeBayes is considered to be haplotype-based as it calls variants based on the literal sequences of reads aligned to a particular target, not their precise alignment. This method avoids nonidentical sequences with multiple possible alignments, which is one of the core problems with alignment-based variant detection.

### Variant quality control and annotation

Individual Variant Calling Format (VCF) files were merged into a single VCF file which was further used for a quality control analysis. Data quality control and variant filtering were performed using VCF tools [28]. Variant filtering consisted of several steps performed in a sequential order as follows: (i) Keeping variants with a minimum quality score of 30 and minor allele count of 3; (ii) Removing all variants with a minimum depth of fewer than three reads; (iii) Removing all individuals with the level of missingness above 0.5; (iv) Removing all variants with a genotype call rate below 0.95 and minor allele frequency below 0.05; (v) Annotating all variants using the ensemble variant effect predictor (VEP) [29] and removing all variants deviating from Hardy-Weinberg equilibrium (*P* < 0.001) (Table S1). Table S1 contains statistics for the above-described filtering steps. The VCF file obtained after performing quality control contained information from 40 individuals and 12,152 genetic variants.

### Genetic association analysis

The VCF file obtained after performing the quality control and filtering of variants (40 individuals and 12,152 genetic variants) was combined with a dataset containing phenotypic information from the 36 participants to compose the final dataset of 36 participants that was used for genetic association analysis. We used the final diagnosis (ASD or other DDs) as an outcome for genetic association analysis. Covariates included child sex, gestational age at delivery (weeks), child birth weight (g), mother’s age at delivery (years), and father’s age at delivery (years) (Table 2). Gene-based association tests, in contrast to variant-based association tests, examine the aggregate effect of both risk and protective variants within a genomic region defined by gene annotations. We conducted gene-based association tests by applying SKAT-O, which optimally combines standard sequence kernel association test (SKAT) and burden tests [30]. Input files for SKAT-O gene-based association analysis were prepared following the instructions from the vignette from the SKAT R package [31]. Gene sets were created based on the VEP annotating genetic variants to their corresponding genes. The final dataset was composed of 36 individuals, with 7101 sets obtained from 22,424 genetic variants.

The power to identify statistically significant associations between genetic variants and target traits decreases as the minor allele frequency decreases. Therefore, the variants with low minor allele frequency are grouped together. The need for grouping genetic variants in this way is generally used in WES and GES studies with small sample sizes. The grouping of genetic variants in the genetic association analysis is performed on the level of the genes by applying collapsing tests. For this purpose, we used the approach that combines burden and non-burden tests [29–32]. This approach has been used in genetic association studies for several different traits, such as myocardial infarction, amyotrophic lateral sclerosis, and blood pressure [33–35].

### Pathway analysis

We performed the pathway analysis using GeneNetwork v2 [31]. GeneNetwork v2 uses gene co-regulation to predict pathway membership and human phenotype ontology (HPO) term associations, as well as other pathway memberships, such as Kyoto Encyclopedia of Genes and Genomes (KEGG), REACTOME, Gene Ontology (GO) including GO cellular component (GO_C), GO molecular functional (GO_F), and GO biological process (GO_P). GeneNetwor v2 implements the GeneNetwork-assisted diagnostic optimization method that can predict phenotypic consequences of genes when mutated, using public RNA-seq data of over 30,000 samples. Using the phenotypes of a patient denoted as HPO terms, GeneNetwork v2 can prioritize genes harboring candidate mutations. The list of 43 highly significant (*P* < 0.001) gene sets was used as input data for GeneNetwork v2. This list was obtained from the gene-based association analysis using the SKAT-O methodology. The list of highly enriched (*P* < 0.001) HPO, GO_C, GO_F, GO_P, and KEGG pathways was obtained (Table S2).

### Quality control

Variant filtering consisted of five quality control steps performed in a sequential order as follows starting: (i) Keeping variants with a minimum quality score of 30 and minor allele count of 3; (ii) Removing all variants with a minimum depth of fewer than three reads; (iii) Removing all individuals with the level of missingness above 0.5; (iv) Removing all variants with a genotype call rate below 0.95 and minor allele frequency below 0.05; and (v) Annotating all variants using Ensemble VEP [27, 28] and removing all variants deviating from Hardy-Weinberg equilibrium (*P* < 0.001) (Table S1). Table S1 contains statistics for the above-described filtering steps, starting with, on average, over 5.8M per genetic variant per sample in the raw VCF files and finishing with an average of over 12K genetic variants per sample after the above five steps.

### Ethical statement

This study was approved by the EDUS Institutional Review Board for the Protection of Human Subjects for all project activities. All participants were recruited through the registered non-governmental organization EDUS-Education for All [19]. The study design and protocol were developed in accordance with the principles of the Helsinki Declaration. Informed written parental consent was obtained for a total of 81 participants out of 126 who were enrolled in the EDUS preschool program during the school year 2018/2019.

## Results

### Participants characteristics

In this sample, 80% of children were males. The mean age of the whole population was 60.1 months (SD ═ 13.4, range 24–85). Mothers’ age at delivery ranged between 18 and 40 years with 61.1% of them being in the 18–30 age bracket and the remaining 38.9% in the 31–40 age bracket. The majority of fathers, 66.7%, were in the age bracket 31–40 years at delivery. Detailed sample characteristics are presented in Table 1.

### Genetic association analysis

In total, 40 participants comprised the final sample size for which whole exome sequencing was conducted. No genome-wide highly significant hits were identified in the entire dataset after adjustment for covariates, but a list of genome-wide suggestive differences. The list of suggestive genome-wide differences (with *P*-value < 0.001) has been identified for *DSE*, *COL10A1*, *DLK2*, *CSMD1*, *FAM47E*, *PPIA*, and *PYDC2* genes (Table 2). The analysis was adjusted for the participant’s sex, birth weight, and gestational age, as well as the mother’s and father’s age at delivery. The whole list of genes with corresponding *P*-values for ASD vs other DD can be found in Table S3.

### Pathway analysis

We identified suggestive differences (*P* < 0.005) for two REACTOME gene sets, four HPO gene sets, four GO_P, four GO molecular functions, four GO_C, and four KEGG gene sets (Table S2a–S2f) respectively.

The REACTOME pathways highly enriched for genes differing between the two groups (ASD and other DDs) are mRNA 3’-end processing and muscle contraction (Table S2a). The KEGG pathways highly enriched for genes differing between the two groups (ASD and other DDs) is endocytosis (Table S2b). HPO terms highly enriched for genes differing between the two groups (ASD and other DDs) are constitutional symptoms, premature ovarian insufficiency, abnormal ovarian physiology, and long eyelashes (Table S2c). GO_Ps highly enriched for genes differing between the two groups (ASD and other DDs) are stem cell population maintenance, response to ionizing radiation, RNA export from the nucleus, and embryonic cranial skeleton morphogenesis (Table S2d). GO molecular functions that are highly enriched for genes differing between the two groups (ASD and other DDs) are lysine-acetylated histone binding, DNA-dependent ATPase activity, histone demethylase activity (H3K9-specific), and proteasome binding (Table S2e). GO_Cs highly enriched for genes differing between the two groups (ASD and other DDs) are the nuclear body, autophagosome, lateral element, proteasome core complex, and beta-subunit complex (Table S2f).

## Discussion

Our exome analysis showed that the following genes differ between ASD and other DDs: *DSE*, *COL10A1*, *DLK2*, *CSMD1*, *FAM47E*, *PPIA*, and *PYDC2*. Some of these genetic risk variants have been previously reported in association with ASD, such as *DSE* and *CSMD1*, while *COL10A1*, *DLK2*, *FAM47E*, *PPIA*, and *PYDC2* have not been reported.

The dermatan sulfate epimerase or *DSE* gene has been associated with Ehlers–Danlos/Hypermobility Spectrum Disorders (EDS/HSD) syndrome. While ASD is primarily defined as a behavioral syndrome, EDS/HSD is a group of hereditary connective tissue disorders believed to be due to a deficiency in collagen fibrillogenesis [36]. However, there are significant phenotypic similarities between these disorders at multiple levels. Both disorders share secondary comorbidities, such as ADHD, anxiety and mood disorders, proprioceptive impairment, sensory hyper/hyposensitivities, eating disorders, epilepsy, suicidality, structural abnormalities, and sleep disorders [36]. Casanova et al. [37] surveyed 702 adults on various EDS/HSD-related health issues, comparing individuals with EDS/HSD, ASD, and unaffected controls. The ASD group reported similar though less severe symptomology as the EDS/HSD group, especially in immune/autonomic/endocrine dysregulation, connective tissue abnormalities (i.e., skin and bruising/bleeding), and chronic pain. Furthermore, EDS/HSD mothers with children with ASD reported more immune symptoms than EDS/HSD mothers without children with ASD, suggesting the maternal immune system could play a heritable role in these conditions. This study implies that EDS/HSD and autism share some aspects of immune/autonomic/endocrine dysregulation, pain, and some tissue fragility, with typically more severe expression in the EDS/HSD [37].

*COL10A1* gene has been associated with age-related macular degeneration, myopia, and refractive error measurement. Age-related macular degeneration (AMD) is a genetically transmitted, neurodegenerative disease that causes central vision loss due to photoreceptor death [38]. Patients with macular degeneration have been observed to have problems with face perception [39]. Seiple et al. investigated if anomalous fixation patterns occur when patients with AMD watch an image of a face. According to their study, patients with AMD had fixation patterns comparable to those seen in other groups of individuals who had difficulty perceiving faces [40]. Individuals with social phobias, Williams syndrome, ASD, schizophrenia, or prosopagnosia, for example, have impaired face perceptions and a considerably higher proportion of fixations on external characteristics of faces [40]. The occurrence of ophthalmologic problems in children with ASD and related conditions was studied by Ikeda et al. [41]. According to their findings, ophthalmologic pathology was discovered in 40% of people with autism or a similar disease, with 29% having refractive errors, 21% having strabismus, and 10% having amblyopia. It suggests that children with autism or related disorders are likely to develop ophthalmologic abnormalities [41].

Another gene found through our analysis is *DLK2* (Delta-like non-canonical Notch ligand 2). The key phenotypes associated with variation in the *DLK2* gene are blood protein levels and waist-to-hip ratio adjusted for BMI [42]. *DLK2* is a newly identified gene that has emerged as a new component in the complex biological processes regulated by *DLK1* and is expressed primarily in the lung, brain, adipose tissue, testes, liver, placenta, ovaries, and thymus [43]. Children with ASD are more likely to be overweight or obese [44]. Corbett et al. [45] compared BMI values in early adolescents with ASD and youth with typical development (TD), finding that pre-to-early puberty children with ASD have higher BMI percentiles than youth with TD, thus implying that they are at higher risk for health issues related to weight.

Some of the most common traits and conditions associated with variation in the *CSMD1* (The Cub and Sushi Multiple Domains 1) gene are PHF-tau measurement, schizophrenia, age at menarche, adolescent idiopathic scoliosis, neurofibrillary tangles measurement, chronotype measurement, and BMI [46]. Hergüner and Hergüner analyzed the relationship between age at menarche and autistic traits in a nonclinical sample of female university students [47]. They estimated that there would be a positive relationship between autistic traits and menarche age because prenatal androgen exposure is involved in the etiologies of both age at menarche and autism. Their results indicate that delayed menarche and autistic traits may share a developmental mechanism, possibly involving high levels of prenatal androgens [47]. Zheng et al. [48] discovered common genetic pathways in schizophrenia and ASD. Many common copy number variations, deletions, and duplications suggest a link between the two illnesses [48]. The fifth gene found to differentiate children with ASD from children with other DDs in this study is *FAM47E*. The key traits and conditions associated with the *FAM47E* gene are Parkinson’s disease (PD), high-density lipoprotein cholesterol measurement, and sleep duration [49]. The *FAM47E* gene has been linked to chronic kidney disease and PD, according to genome-wide association studies [49]. Autism and PD are two very different disorders that appear to have little in common other than the fact that they are both brain disorders. Both conditions, however, appear to be genetic with varied penetrance (though autism’s penetrance may be much higher), and both seem to be highly susceptible to environmental factors [50]. Repetitive behaviors of a compulsive–impulsive type can be present in both disorders, with it being one of three core symptoms required for the ASD diagnosis [50]. In a study of middle-aged and older persons, Starkstein et al. [51] looked at the link between Parkinsonian symptoms and ASD. They conducted an exploratory investigation on 19 people with ASD who were 49 years or older in the pilot study (study I). In the second part of the study, the authors investigated the hypothesis that “older” people with ASD have higher frequencies of Parkinsonian symptoms [51]. The outcomes of this study reveal that people with ASD beyond the age of 39 had a significant risk of Parkinsonism [51].

The last two genes found in this study to differentiate between ASD children and other DD children are *PPIA* and *PYDC2*. The main trait associated with or affected by the variation within the *PPIA* gene is intelligence [52]. A study by the Centers for Disease Control and Prevention (CDC) reported that among 3.604 children with ASD included in their sample, 31% had been classified in the range of intellectual disability, and 23% were in the borderline range [53]. Among children identified with ASD who had IQ scores available, about one-third (35.2%) also had intellectual disability [54].

A limitation of our exploratory study is that we had a very small cohort of diagnosed ASD children and other DDs. However, we used collapsing tests to group genetic variants, combining burden and non-burden tests. Larger replication studies are still needed to confirm our findings. Furthermore, we could not expand our follow-up search to non-coding DNA regions in this exploratory study; it should be considered in future follow-up studies. Incorporating sequencing and genotyping methodologies that will cover the non-coding regions of the genome in the future replication study will be needed. We also did not have parents sequencing data to perform a trio analysis and understand whether these are somatic or germline genetic variants. Future replications would benefit from trio studies to determine the distribution of somatic and germline variants.

Since subjects included in this discovery cohort may not be representative of the whole ASD and other DD populations, the generalizability of findings from the current study may be limited. Therefore, selective convenient sampling could potentially be the source of bias in the analysis. However, all children obtained clinically confirmed diagnoses based on DSM V (2013) [55], and are similar to children with the same diagnosis, not included in the analysis.

As the prevalence of ASD is constantly rising and is at its peak of 1 in 36 children being diagnosed [53], there is a growing concern about diagnosis strategies and improving the quality of life. The advent of genetics has brought novel insights that can potentially be used for early screening purposes. These tests can be developed by selecting the genes and whose mutation patterns differ in ASD and other DDs, thereby enabling differentiation between these groups.

## Conclusion

In this exploratory study, we performed genetic association analysis using whole exomes of 36 children with ASD and other DDs. This exploratory study is one of the first to explore the genetic difference between individuals with ASD and other DDs. The analysis yielded seven suggestive gene candidates (*DSE*, *COL10A1*, *DLK2*, *CSMD1*, *FAM47E*, *PPIA*, and *PYDC2*) to differentiate observed phenotypic characteristics between our cohort participants with ASD and other DDs. Pathway analysis revealed several biological processes to be distinct between ASD and other DDs; however, firm conclusions are difficult to draw because of the lack of replication studies in large samples. Our genetic results might be of added value to differentiate individuals with ASD from other DDs, but future analyses in a larger population are needed to confirm this hypothesis. Furthermore, it would be useful to explore whether information from this exploratory study may add predictive value on top of known risk factors for ASD and other DDs. Given the identified effect sizes were small, so it is unlikely that information on single genetic variants are sufficient to predict and distinguish ASD from other DDs, as complex disorders. However, these variants might improve polygenic risk scores and help improve predictive power, capturing broader patterns distinguishing ASD and other DDs early.

## Supplemental data

**Supplementary methods 1:** https://www.bjbms.org/ojs/index.php/bjbms/article/view/10221/3304

**Supplementary methods 2:** https://www.bjbms.org/ojs/index.php/bjbms/article/view/10221/3305

**Table S1:** https://www.bjbms.org/ojs/index.php/bjbms/article/view/10221/3306

**Table S2:** https://www.bjbms.org/ojs/index.php/bjbms/article/view/10221/3307

**Table S3:** https://www.bjbms.org/ojs/index.php/bjbms/article/view/10221/3308

## Data Availability

The datasets generated during and/or analyzed during the current study are available from the corresponding author on reasonable request.
